# Gap formation and dynamics after long‐term steady state in an old‐growth *Picea abies* stand in Norway: Above‐ and belowground interactions

**DOI:** 10.1002/ece3.3643

**Published:** 2017-11-30

**Authors:** Per Holm Nygaard, Line Tau Strand, Arne Oddvar Stuanes

**Affiliations:** ^1^ Norwegian Institute of Bioeconomy Research Aas Norway; ^2^ Department of Environmental Sciences Norwegian University of Life Sciences Aas Norway

**Keywords:** biomass decline, gap initiation, late‐successional dynamics, long‐term permanent plots, nutrients

## Abstract

Stand dynamics and the gap initiation prior to gap formation are not well‐understood because of its long‐term nature and the scarcity of late‐successional stands. Reconstruction of such disturbance is normally based on historical records and dendroecological methods. We investigated gap initiation and formation at the fine‐scale stand level in the old‐growth reserve of Karlshaugen in Norway. Given its long‐term conservation history, and thorough mapping in permanent marked plots with spatially referenced trees, it provides an opportunity to present stand development before, during, and after gap formation. Late‐successional decline in biomass was recorded after more than 50 years of close to steady state. Gaps in the canopy were mainly created by large old trees that had been killed by spruce bark beetles. Snapping by wind was the main reason for treefall. Long‐term dominance of Norway spruce excluded downy birch and Scots pine from the stand. Comparisons of the forest floor soil properties between the gap and nongap area showed significantly higher concentrations of plant available Ca within the gap area. Plant root simulator (PRS™) probes showed significantly higher supply rates for Ca and Mg, but significantly lower K for the gap compared to the nongap area. Soil water from the gap area had significantly higher C:N ratios compared to the nongap area. Fine‐scale variation with increasing distance to logs indicated that CWD is important for leaking of DOC and Ca. Our long‐term study from Karlshaugen documents gap dynamics after more than 50 years of steady state and a multiscale disturbance regime in an old‐growth forest. The observed disturbance dynamic caused higher aboveground and belowground heterogeneity in plots, coarse woody debris, and nutrients. Our study of the nutrient levels of the forest floor suggest that natural gaps of old‐growth forest provide a long‐lasting biogeochemical feedback system particularly with respect to Ca and probably also N. Norway spruce trees near the gap edge responded with high plasticity to reduced competition, showing the importance of the edge zone as hot spots for establishing heterogeneity, but also the potential for carbon sequestration in old‐growth forest.

## 1 INTRODUCTION

Old‐growth forests are characterized by the presence of large trees and complex horizontal and vertical structures and large amounts of woody detritus (Harmon et al., 1986; Lewis & Lindgren, 2000; Oliver & Larson, 1996; Wirth et al., 2009; Messier, Bergeron, Frank, & Fankhanel, 2009). In Fennoscandia, southern old‐growth boreal forests have almost disappeared, mainly because of clear‐cutting (Ostlund, Zackrisson, & Axelsson, 1997). In Norway, about 1% of the forest cover can be regarded as old growth and these forests are mainly located in protected areas (Tomter & Dalen, 2014). Old‐growth forests provide a range of ecosystem services including biodiversity preservation, cultural services, and carbon sequestration. The potential for carbon sequestration in old‐growth and unmanaged forests has been disputed (Luyssaert et al., 2008; Nabuurs et al., 2013), and the exclusion of unmanaged old‐growth forests from the Kyoto protocol due to the perception of late‐successional biomass production has been questioned (Freibauer, 2009; Schulze et al., 2009).

Previously, old‐growth forests were regarded as a stable climax community (Cajander, 1926; Odum, 1969; Weaver & Clements, 1938). Sernander (1936) and (Watt, 1947) emphasized disturbance as an important process in old‐growth forest; however, it was not until the 1970s that this view was widely accepted (Borman & Likens, 1979; Heinselman, 1970; van der Maarel, 1996; Sprugel, 1976). Large‐scale stand‐replacing calamities such as fire and windthrow have been regarded as the most important disturbance factors in boreal forests (Sirén, 1955; Zackrisson, 1977). However, if stand‐replacing calamities have long return intervals compared to the longevity of the tree species, a new small‐scale dynamic equilibrium driven by intrinsic tree population processes will develop. The importance of small‐scale gap or patch dynamics in old‐growth boreal forests has probably been underestimated compared to large‐scale disturbances (Kuuluvainen, Syrjanen, & Kalliola, 1998; McCarthy, 2001). Descriptions of gaps and gap dynamics are presented in a number of papers based on different methods, terminology, and traditions (Kuuluvainen, 1994; Kuuluvainen & Aakala, 2011; McCarthy, 2001; Schliemann & Bockheim, 2011; Schulze, Wirth, Mollicone, & Ziegler, 2005; White & Jentch, 2001). Disturbance regarded as a gap or stand‐replacing dynamic, or small‐scale cycle or large‐scale cycle, has been questioned (Kneeshaw, Bergeron, & Kuuluvainen, 2011; Kuuluvainen, 2009; Kuuluvainen et al., 2014; Worrall, Lee, & Harrington, 2005). The conceptual dichotomy seems to be an oversimplification of a far more complex temporal and spatial disturbance dynamic. Worrall et al. (2005) proposed a system of gap phase disturbance cycles nested within larger episodic disturbance. A study of old‐growth Norway spruce (*Picea abies*) on a landscape scale from Archangelsk demonstrated a multiscaled disturbance regime with a combination of small stand‐scale gap dynamics, and landscape scale intermediate severity mortality episodes (Kuuluvainen et al., 2014). Kuuluvainen and Aakala (2011) reviewed natural forest dynamics in boreal Fennoscandia and concluded that only two of 48 papers discussed old‐growth *Picea abies* forests from the southern boreal zone. Few studies have documented gap initiation or identified the disturbance factors that cause the death of gap‐maker trees (Worrall et al., 2005).

In this study, we present data on stand‐scale disturbance history from the Karlshaugen reserve which has been intensively monitored since 1930 and gap formation which has developed during the last decades. The stand in question differs from the other stands in the reserve by a high standing volume and gap formation after 50 years of near steady‐state behavior. The presented study is a small‐scale case study describing the stand dynamics and patterns along gap formation, but we believe this study reveals generality, as the forest vegetation of Karlshaugen represents the most common vegetation type over large portions of the European boreal forests. We used a combination of repeated measurements in permanent plots, dendroecological techniques, and GIS. Our main objective was to assess whether the aboveground biomass (AGB) was in steady state prior to gap initiation and to document the gap formation by examining gap‐maker trees and the gap created. Our second objective was to assess the differences in which elements and compounds are lost and retained in the gap area compared to the nongap areas 20 years after gap formation. We used a combination of soil sampling, lysimeter techniques, and plant–root simulators. Finally, we discuss the observed disturbance regime in light of ecological theory and implications for forest management.

## 2 METHODS

### 2.1 Study area

**Figure 1:**
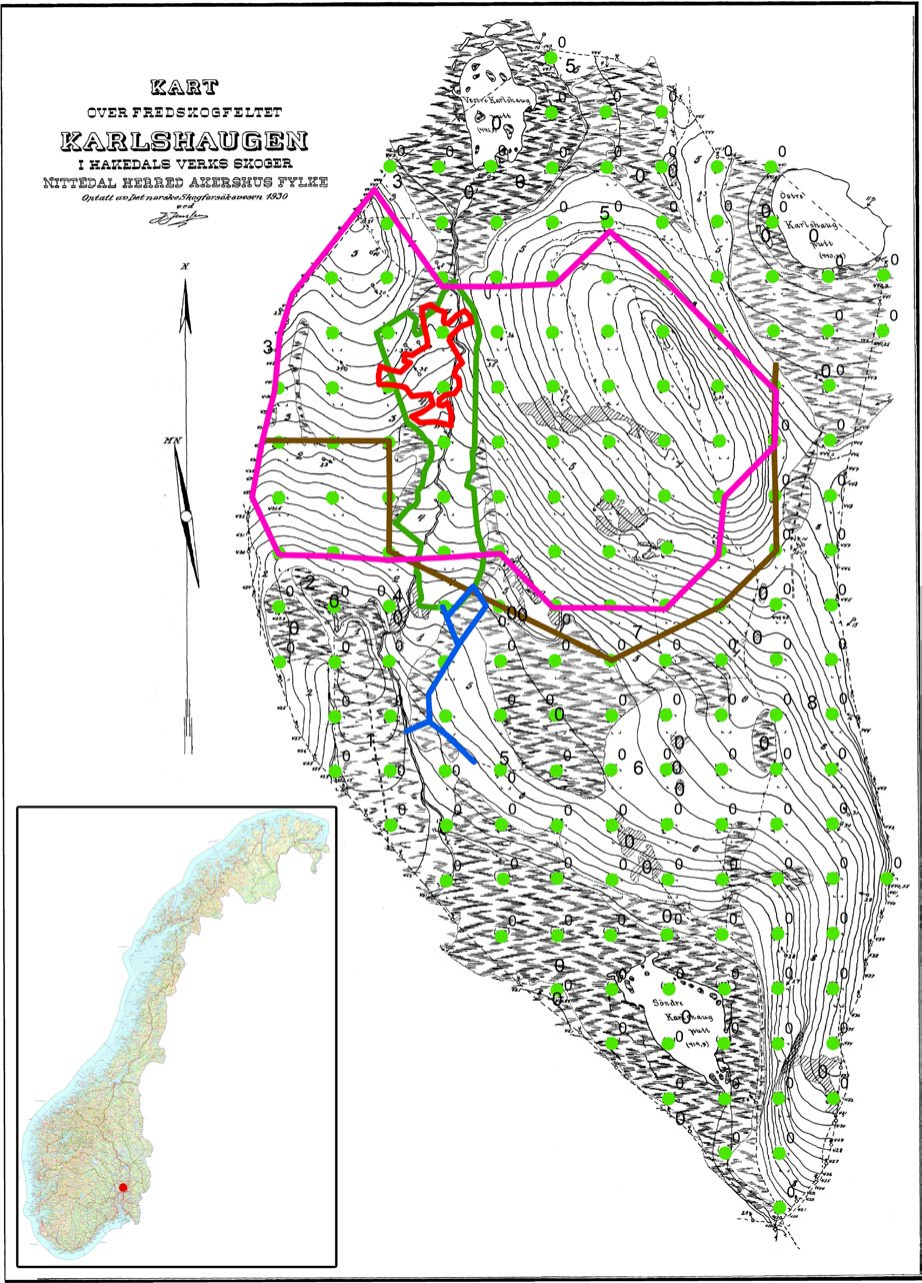
The location of the nature reserve in Norway. The old map from 1930 showing the grid plots (green dots), the stand polygon (green), the gap polygon (red), the fire polygon (purple), and demarking line for selection cutting (brown) and ditching (blue)

Karlshaugen is a 15 ha nature reserve, which has been conserved since 1922, and is located in the south boreal zone (Moen, 1998). It is situated 20 km north of Oslo, Norway (10°48′E, 60°05′N), at 420–450 m above sea level (Figure 1). The Karlshaugen reserve consists of 9.8 ha of forest land, 4.4 ha of peat land, 0.5 ha of open water, and 0.3 ha of bedrock (Braathe, 1980). In 1930, the area was mapped, and 171 permanent grid plots, 5 × 10 m in size, were established in a 30 × 30 m grid covering 5.6% of the area (Figure 1). Numbered iron posts in the NW corner marked the location of the grid plots. The mean annual precipitation is 1,200 mm, of which 417 mm falls in May to August, and the mean annual temperature is 3.4°C, with a mean temperature of 12°C for May to August, according to the 1960–1991 temperature normal. The main tree species are Norway spruce (*Picea abies* (L.) H. Karst), Scots pine (*Pinus sylvestris* (L.), and downy birch (*Betula pubescens* (Ehrh.). The ground vegetation is dominated by bilberry (*Vaccinium myrtillus* L.) and can be assigned to the *Eu‐Piceetum* and *Vaccinio‐pinetum* associations (Kielland‐Lund, 1994). The species richness of the reserve is rather low with 85 vascular plants, 69 bryophytes, and 27 lichens which been recorded (Lid, 1960; Nygaard & Odegaard, 1999). Vegetation analysis carried out in permanent plots at Karlshaugen from 1931, 1961, and 1991 showed high stability at the stand level, but also high dynamics at a finer scale (1 m^2^) (Nygaard & Odegaard, 1999). Photographs were taken from 40 fixed positions in 1930, 1978, 1997, and 2013. The ages of the forest stands vary from 150 to 300 years, with a mean age from breast height for dominant Scots pine and dominant Norway spruce of 269 and 178 years, respectively. Dominant trees are defined as the 10 largest trees per hectare. The soils in the area vary with well‐developed Podzols, on short transported shallow‐till deposits, merging into Gleysols and Histosols in more poorly drained areas around the three small forest ponds (Figure 1).

### 2.2 Disturbance history

In the process of establishing the Karlshaugen nature reserve in 1922, the forest was described as pristine where no cutting had taken place within “the memory of man” (Braathe, 1980). However, a thorough mapping of felled stumps in 1930 showed that dimension cutting had taken place around 1880 in the southern part of the area (unpublished data by J. Jensen 1930), while no stumps were recorded in a smaller area north of the demarking line where “the special” stand analyzed in this study is situated (Figure 1). A few fire‐scarred dead pine trees and stumps were documented in 1930, and charcoal was frequently observed in the soil. Tree‐ring cross‐dating of fire scars from 2014 against the chronology of Eidem (1959) suggested that the last fire took place in 1761. Some small‐scale draining by ditching and restoration of a brook was carried out in 1913, resulting in a ditch length of 173 m, which has influenced parts of the area (Figure 1). However, the most common disturbance agent in this area is snow break, and many trees have been broken repeatedly (Braathe, 1980).

### 2.3 The stand and the gap

**Figure 2:**
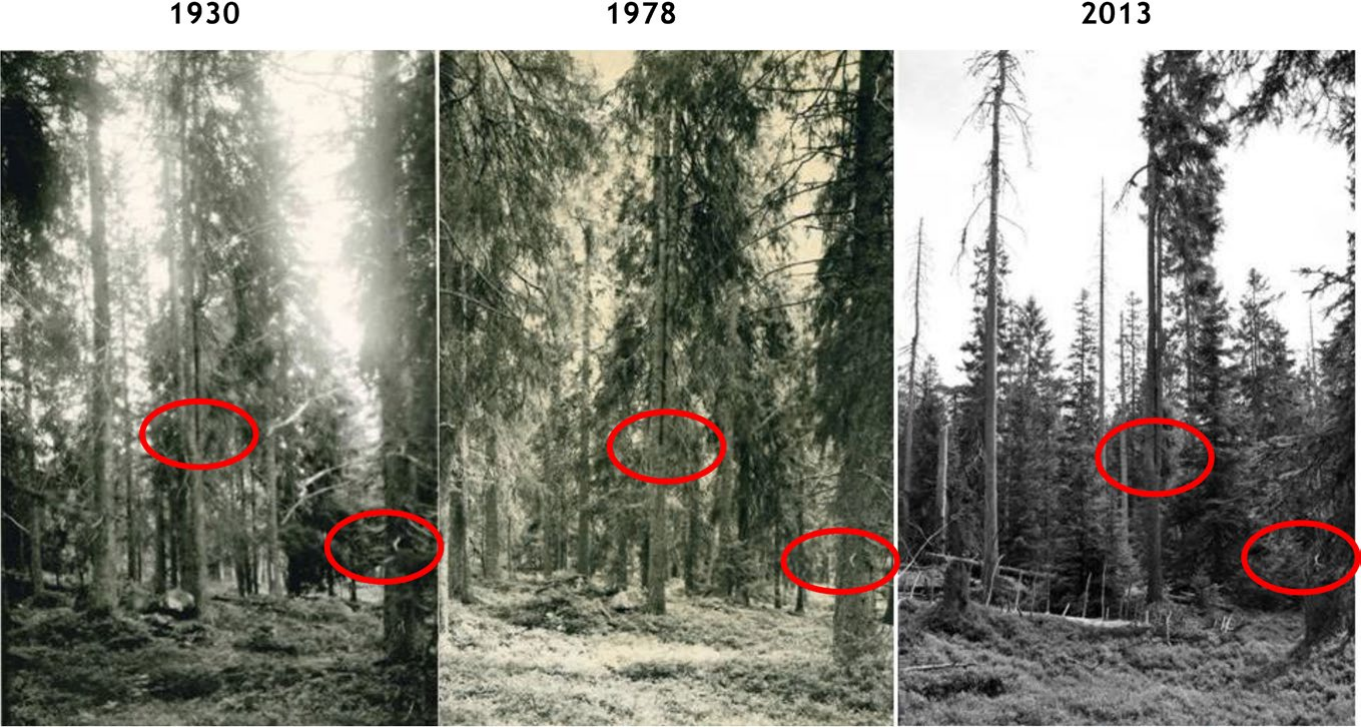
Part of the gap area photographed before and after gap formation. The double top tree in the middle (red) of the photograph is the same tree. The tree close to the right‐hand edge of the picture is snapped, but the dry twig on the stem and on the snag can be seen on all photos. Note the dominance of *Vaccinium myrtillus* at all point of time

The stand with the gap formation is a 0.57 ha Norway spruce stand with a few large scattered Scots pines and is situated within the least anthropogenically disturbed area (Figure 1). If not otherwise stated, the results presented in this work are restricted to this stand. The ground vegetation is dominated by *V. myrtillus*,* Vaccinium vitis‐idea (L.)*,* Majanthemum bifolium* (L.), *Dicranum majus* (Sm.), *Dicranum scoparium* (Hedw.), *Hylocomium splendens* (Hedw.) Schimp., *Sphagnum girgensohnii* (Russ.), and *S. capillifolium* (Ehrh.)Hedw. Six grid plots of 50 m^2^ are located inside the stand. Mean age at breast height for Norway spruce, based on coring of 15 trees of different diameter in 2014, showed 175 years (*SD* = 38) (Figure 7). Charcoal recorded from soil samples in 2014 showed that a larger area including the actual stand has burned. No radiocarbon dating of charcoal was carried out; however, from the age of the stand and dating of fire scars, we assume that the 1761 fire was a stand‐replacing fire. The first sign of gap initiation was observed in 1993 (Nygaard & Odegaard, 1999), the gap area is shown as a red “Donald” shaped polygon within the stand map (Figure 1.) The gap formation was initiated by bark beetles (*Ips typographus* L.) which attacked living trees in 1993, a peak year according to the bark beetle monitoring program of Norway (http://www.skogoglandskap.no/temaer/barkbilleovervaking). Even though the trees were attacked in 1993, there was some time lag between the attack and mortality. During 1994–1996, most of the damaged trees were snapped by wind. Tipping of root plates was not recorded even if uprooting often is reported during gap formation (Bauhus, 2009; Hytteborn & Verwijst, 2014; Kauhanen, 1991; Sernander, 1936; Sirén, 1955). Nine scattered living trees are still standing inside the gap, and 10 dead trees are seen outside the margin of the gap (Figure 5). No expansion has been observed after 1996. So far, this is the only gap formation within the small area with little human impact. Photos taken from a fixed position close to the gap show the forest structure before and after gap formation (Figure 2).

### 2.4 Sampling and analyses

In 1930, height and diameter at breast height (DBH) of all stems with DBH >2.0 cm were recorded for all grid plots in the entire reserve. Spatial position within the grid plots was recorded for all registered stems. The grid plots were reanalyzed in 1978, 1997, and 2013 by the same methods. A planned revision of grid plots in 1955 was canceled because the field books were misplaced, but fortunately recovered later. In addition, a complete inventory of DBH for every living stem larger than 2.0 cm in each stand was carried out in 1930, 1955, 1978, and 1997. DBH measurements were obtained using calipers and height measurements using a measuring rod and theodolite, or by a Vertex hypsometer in the last inventories. The volume of living trees and standing dead trees on the grid plots was calculated using volume equations for each tree species based on DBH and height (Braastad, 1966; Brantseg, 1967; Vestjordet, 1967). Aboveground biomass for Norway spruce on the grid plots was calculated according to Lehtonen, Makipaa, Heikkinen, Sievanen, and Liski (2004). A complete inventory of downed dead wood inside the gap area was recorded by measuring diameter (DBH) and length in 2001. Downed dead wood within the grid plots was recorded in 2013 by measuring midpoint diameter and length inside the grid plots for logs with diameter larger than 10 cm. Advanced growth and saplings higher than 10 cm within the gap area were recorded, and height of each plant was measured to the nearest decimeter in 2001.

Collection of tree‐ring data was restricted due to conservation rules. The data consist of single increment cores from 10 dominant canopy trees of Norway spruce to 10 dominant canopy trees of Scots pine for determining the age at breast height of the forest in the entire reserve. The dominant trees were subjectively sampled among the largest diameter trees with no damages. Fifteen trees of Norway spruce of different DBH were cored within the stand for determining age and response to gap formation. In addition, twenty individuals of suppressed advanced growth within the fire polygon (Figure 1) were cut near the surface for determination of age. The cores were wetted and prepared by scalpel, and zinc pastes before annual ring width were measured by an Addo micrometer. In cases of missing pit, the missing rings were estimated based on curvature and width of the innermost ring (Arno & Sneck, 1977).

Cross‐dating of fire scars against the Flesberg chronology (Eidem, 1959) was carried out by the program COFECHA (Holmes, 1994). The old map was digitized and geo referenced to UTM 32, WGS 84 in ARCGIS. The gap area was described and analyzed from an aerial photo using a digital photometric 3‐D workstation and the software Microstation.

### 2.5 Soil water

Soil water from the O horizon was sampled at 16 sites: 10 of which were located within the gap area and six were within the nongap area. In addition, two gap‐maker logs were chosen for a more detailed study of soil water beneath decaying wood. Here, samples were taken in the O horizon directly beneath the log and at 10 and 90 cm distances from the log in two directions. Soil water samples were collected on seven occasions from October 2008 to September 2009, with no sampling from November 2008 to May 2009.

Soil water was extracted using lysimeters of the macro–rhizon type (type 19.21.35 Eijkelkamp, the Netherlands). These were installed in the O horizon (5–10 cm depth) with three lysimeters at each sampling site. Fifty‐milliliter syringes were used to extract the soil water through the lysimeters. The soil water samples from the three lysimeters were bulked as one sample. Depending on the weather prior to the sampling, the lysimeters yielded between 0 and 50 ml (in average 15 ml) at each collection. The samples were stored cold (<4°C) until analysis.

Dissolved organic carbon was determined using a total organic carbon analyser (TOT‐V CPN Shimadzu) according to NS 1484 (NSF, 1997). Total nitrogen (Tot‐N) was determined photometrically (Flow injection analysis FIA star 5020 analyser, Tecator) after oxidation with peroxodisulphate following Norwegian standard NS4743 (NSF, 1993). Ammonium‐N (NH_4_‐N) was determined photometrically (Gilford Instruments) according to Norwegian standard NS4746 (NSF, 1975a) while Nitrate‐N (NO_3_–N) was determined photometrically (Flow injection analysis FIA star 5020 analyser, Tecator) according to NS4745 (NSF, 1975b). Dissolved organic nitrogen (DON) was calculated as Tot‐N minus the sum of NH_4_–N and NO_3_–N. pH was determined potentiometrically using a glass membrane combination electrode (ORION SA 720 pH/ISE meter). Selected cations (Mg, Ca, and K) were analyzed using inductively coupled plasma mass spectrometry (ICP‐MS; Agilent 8800 Series Triple Quad).

### 2.6 Soil

Soil samples were taken from the stand at each of the sampling sites when the lysimeters were dismantled. Only samples of the O horizon were taken. Half of each soil sample was dried, the remaining fresh soil samples were stored cold (<4°C) until analysis. Inorganic nitrogen components were extracted from the fresh soil samples by extraction with 2 mol/L KCl, the NH_4_–N, and NO_3_–N in these extracts were analyzed as described for the water samples above. The dried samples were sieved trough a 2‐mm sieve. Total carbon (Tot‐soil C) and total nitrogen (Tot‐soil N) were measured by dry combustion using a LECO TruSpec CNH analyser. Soil pH was measured in a 1:2.5 v/v ratio of soil to distilled water using a glass membrane combination electrode (ORION SA 720 pH/ISE meter). Selected cations (Mg, Ca and K) were extracted by 0.4 mol/L acetic acid and 0.1 mol/L ammonium lactate at a pH of 3.75. These cations were analyzed using inductively coupled plasma optical emission spectrometry (ICP‐OES; Perkin Elmer Optima 3000 DV).

### 2.7 Plant and root simulator (PRS) probes

PRS™‐probes (Western Ag Innovations Inc., Saskatoon, SK, Canada) were installed close to each of the 16 soil water sampling sites. In addition, PRS™‐probes were installed at 24 randomly chosen sites, within homogenous bilberry vegetation and avoiding coarse woody debris; 10 in the nongap area and 14 in the gap area. The PRS™‐probes were installed in pairs (anion and cation exchange), with four pairs at each installation. Each PRS™‐probe was inserted into the O horizon slightly angled to ensure good contact with the soil and to ensure that the entire membrane was within the O horizon. The 16 PRS™‐probes at the lysimeter sites were left in the soil for 9 weeks, while the 24 randomly placed PRS™‐probes were left for a period of 16 weeks. After retrieval, each PRS™‐probe was rinsed free of adhering soil with de‐ionized water. All PRS™‐probes located on the same site were put in the same zip lock bag and kept cool and moist until they were sent to Western Ag Innovations Inc., Saskatoon, SK, Canada. There the PRS™‐probes were eluted using a 0.5 N HCl solution for 1 hr. NO_3_–N and NH_4_–N in the eluate were analyzed by colourimetry using an automated flow injection analysis (FIA) system. Inductively coupled plasma (ICP) was used to analyze the Ca, Mg, and K of the PRS™ eluate. Only NO_3_–N and NH_4_–N were analyzed for the 16 PRS™‐probes from the lysimeter sites, while a complete analysis of both NO_3_–N and NH_4_–N and other plant nutrients was analyzed for the 24 random sample sites. Only supply rates (micrograms/10 cm^2^/burial length) of NO_3_–N, NH_4_–N, Ca, Mg, and K are reported in this study.

### 2.8 Charcoal registration

In each grid plot within the stand, two soil samples were collected close to the NW corner using a cylindrical auger of 5 cm in diameter and 45 cm in length. Macroscopic charcoal (≥0.5 mm) was recorded by visual inspection using a binocular within a laboratory (Zeiss Stereomicroscope, Stereo Discovery.V20, Carl Zeiss Microimaging GmbH, Göttingen, Germany). In addition, charcoal was recorded eastward and southward for the stand. Based on this sampling scheme, fire scars, earlier soil sampling, and descriptions a convex fire polygon were established (Figure 1).

## 3 RESULTS

### 3.1 Stand and gap dynamics

Stand characteristics based on the six grid plots for the investigation period 1930–2013 are presented in Table 1. An accumulated standing volume slightly larger than 350 m^3^/ha and an aboveground biomass (AGB) of 179 t/ha was recorded for the stand in question in 1930. The standing volume is regarded as maximum for this site index according yield tables for Norway (Braastad, 1975). Over the following 65‐year period, up until gap formation, the stand seems to have been close to steady state with respect to biomass on plot scale.

**Table 1:** The volume and number of living and dead spruce, pine, and birch trees (DBH > 2 cm) based on the grid plots from the stand for each registration. In addition, aboveground biomass for spruce and downed coarse woody debris for 2013 is presented. Standard deviations are in parentheses

	1930	1978	1997	2013
Norway spruce				
Volume of living trees (m^3^/ha)	287 (139)	294 (199)	207 (153)	205 (94)
Number of living trees (per ha)	1,200 (219)	933 (432)	700 (374)	733 (301)
Volume of dead trees (m^3^/ha)	5 (7)	28 (65)	96 (141)	99 (126)
Number of dead trees (per ha)	200 (178)	267 (301)	367 (344)	433 (344)
Scots pine				
Volume of living trees (m^3^/ha)	10 (23)	0	0	0
Number of living trees (per ha)	33 (81)	0	0	0
Volume of dead trees (m^3^/ha)	4 (8)	0	0	0
Number of dead trees (per ha)	67 (103)	0	0	0
Birch				
Volume of living trees (m^3^/ha)	66 (103)	0	0	0
Number of living trees (per ha)	33 (82)	0	0	0
Volume of dead trees (m^3^/ha)	1 (2)	3 (7)	0	0
Number of dead trees (per ha)	33 (82)	33 (82)	0	0
Aboveground biomass trees (t/ha)	179	178	123	122
Downed coarse woody debris (m^3^/ha)	—	—	—	106 (133)

—, Not analyzed.

**Figure 3:**
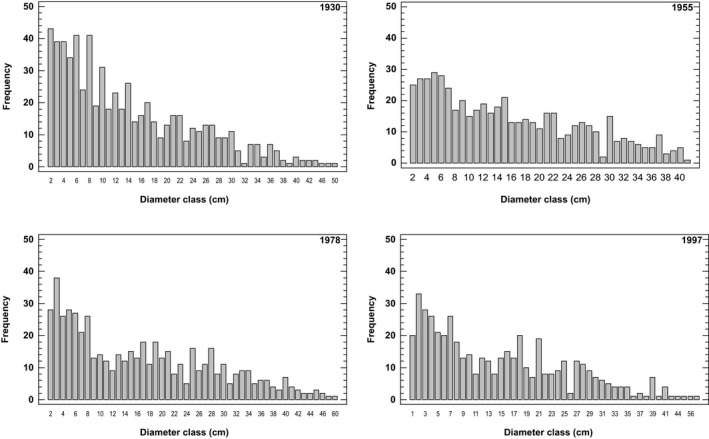
Diameter class (cm) distributions for all living Norway spruce stems in the stand at the four separate inventories

This indication of steady state is supported by the complete inventory of diameter classes of Norway spruce from the stand in 1930, 1955, 1978, and 1997. Frequency distributions of diameter at breast height for all trees >2 cm are shown in Figure 3. The diameter class distributions in cm from 1930, 1955, 1978, and 1997 were tested for significant differences using the Kolmogorov–Smirnov test (Conover, 1980). No differences were found between 1955, 1978, and 1997 indicating a steady state for this period. However, the 1930 distribution was significant different from 1955 distribution (K‐S statistic = 2.06, *p* = .00038), the 1978 distribution (K‐S statistics = 2.48, *p* = .0000009), and the 1997 distribution (K‐S statistics = 1.92, *p* = .0011). The total number of stems from the complete inventories of the stand was 650 in 1930, 540 in 1955, 546 in 1978, and 479 in 1997.

**Figure 4:**
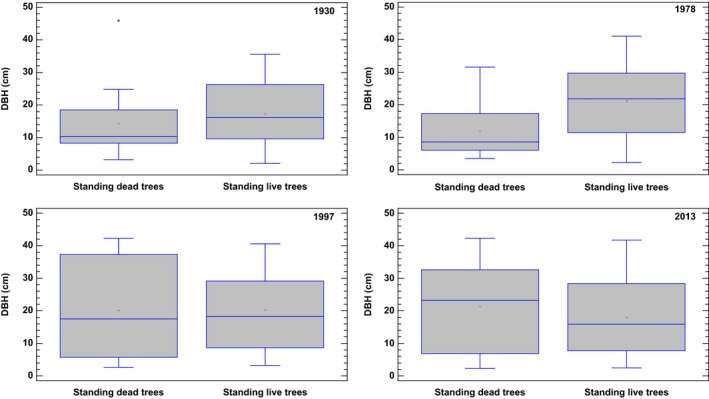
Boxplots of DBH of dead and live trees on the grid plots in the stand at each point of time. The box encloses the middle 50% of observations. Median is represented as a vertical line inside the box and +display the mean. The upper whisker is drawn from upper quartile to the largest observation, while the lower whisker is drawn from the lower quartile to the smallest observation

Measurement and remeasurement from the grid plots in 1930 and 1978 showed a smaller median DBH for dead trees compared to living trees (Figure 4).

DBH of living trees depends on ingrowth and mortality. Median DBH of living trees increased from 1930 to 1978 showing increment exceeded ingrowth and mortality (Figure 4). The remeasurement in 1997 showed equal DBH of living and dead trees, and finally, in 2013, the DBH of living trees was smaller compared to dead trees.

**Figure 5:**
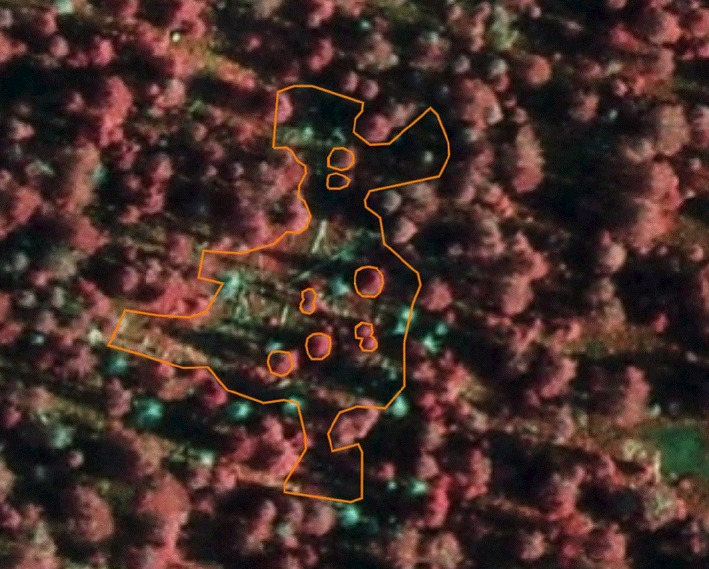
The “Donald” shaped gap area with nine living trees still standing inside the gap area and 10 dead standing trees (green) at the gap margin

The trees fall down during 1994 and 1996 leaving a “Donald” shaped gap with an area of 1,161 m^2^ and a gap volume of 23,220 m^3^ after correcting for nine crown polygons of living trees inside the gap area (Figure 5). The ortho photograph is an infrared (IR) photography of the gap showing living trees in red color and dead trees in green color.

**Figure 6:**
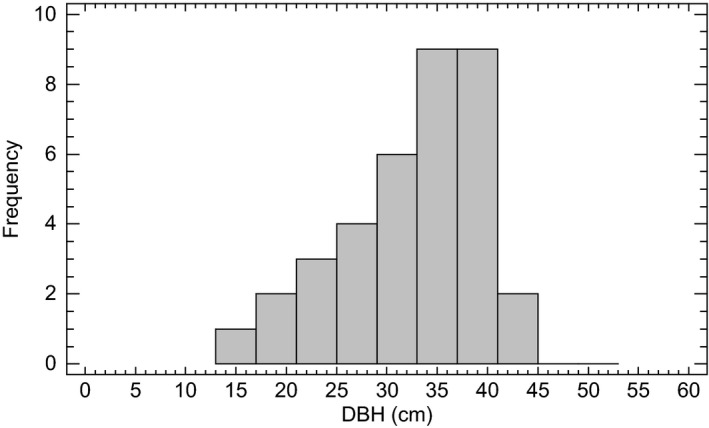
Frequency distribution of stem diameter (DBH) of the gap‐maker trees recorded within the 1,161 m^2^ gap area

**Figure 7:**
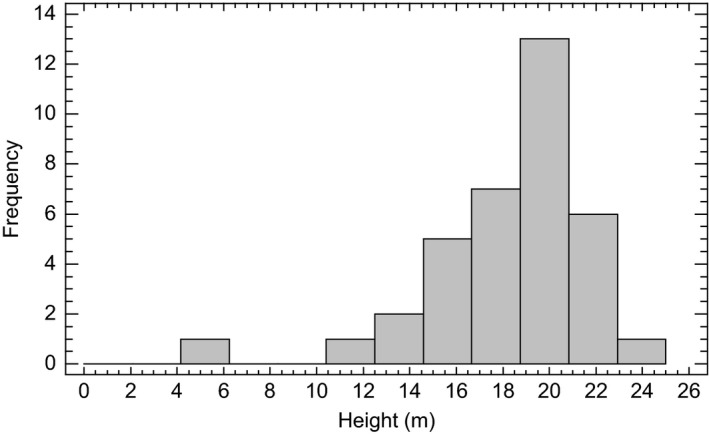
Frequency distribution of height of the gap‐maker trees recorded within the 1,161 m^2^ gap area

The distribution of diameter and height of the gap‐makers is shown in Figures 6 and 7. Gap‐maker trees were with few exceptions dominant trees with diameter and height in the upper end of the scale. The few smaller trees were probably hit by larger treefall.

**Figure 8:**
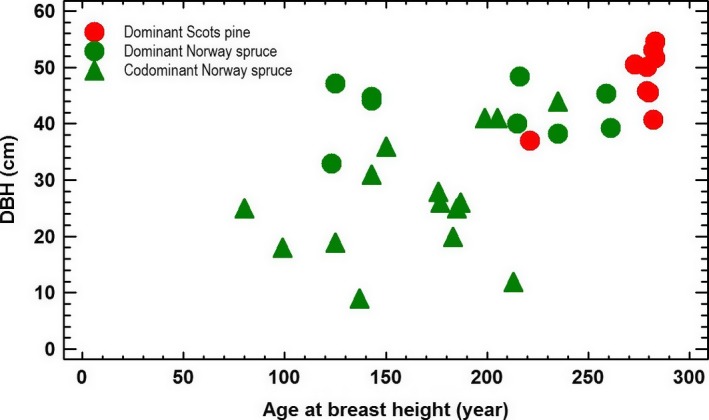
DBH and age at breast height for dominating Scots pine and dominating Norway spruce within the reserve. Triangles show DBH and age at breast height for codominant Norway spruce within the stand

Ages at breast height of dominant Scots pine and Norway spruce from the entire reserve together with 15 Norway spruce from the stand are presented in Figure 8. Circles representing dominant trees from the entire reserve showing Scots pine reaching close to 290 years age at breast height while dominant Norway spruce vary from 130 to 260 years at breast height. For the special stand in question, the age at breast height for Norway spruce vary from 80 to 240 years. A smaller tree with diameter (DBH) 12 cm showed an age at breast height at 213 years.

**Figure 9:**
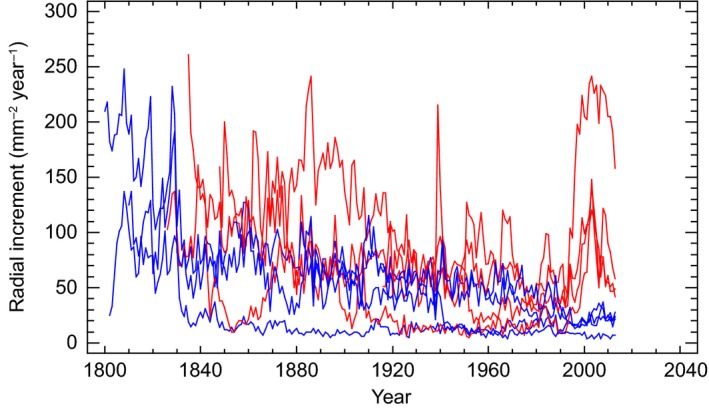
Tree‐ring widths of trees close to the gap edge (red) increased in 1996 compared to those from trees at a distance more than 20 m away from the gap edge (blue)

Response of gap formation on tree‐ring widths of selected trees at different distance to gap edge is shown in Figure 9. Old Norway spruce trees close to the gap edge showed increased radial growth compared to trees more than 20 m away from the gap edge. The growth reaction in 1996 concurs fairly good with the gap formation.

**Figure 10:**
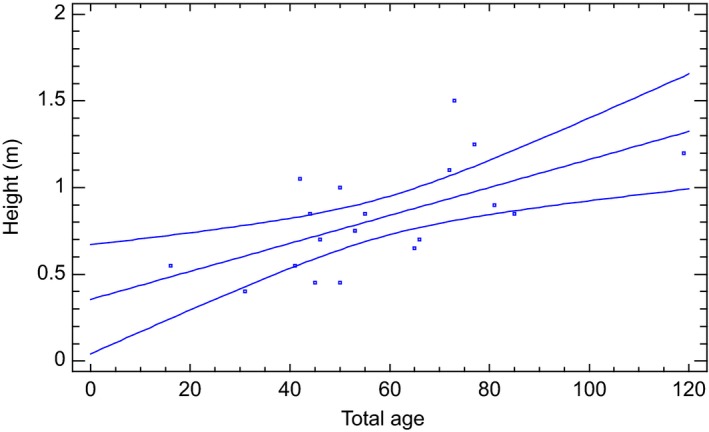
Height versus total age of suppressed advanced growth of Norway spruce collected from the fire polygon, green lines showing 95% confidence limits

Suspended advanced growth of Norway spruce collected from the stand and fire polygon in 2001 showed that time for reaching breast height is about 60–80 years (Figure 10). One of the supended trees is shown to use 119 years for reaching a hight of 1.2 m.

The total amount of downed coarse woody debris (CWD) within the gap was 240 m^3^/ha. Downed coarse woody debris from the permanent plots in 2012 showed a mean of 106 m^3^/ha.

The regeneration, including advanced growth, was analyzed for the gap in 2001, and 9,681 saplings per ha with a mean height of 30 cm were recorded. The field layer in the gap showed only small changes; establishment of *Avenella flexuosa*,* Deschampsia cespitosa,* and some scattered individuals of *Betula pubescens* and *Sorbus aucaparia* were observed, but the gap area was still dominated by bilberry.

### 3.2 Forest floor 20 years after gap formation

There were no significant differences between the forest floor soil properties (Table 2) in the gap and outside the gap with respect to C and N concentrations. However, the results of the soil water analysis suggest that there were differences between the gap and nongap areas with respect to C and N dynamics. There were significantly higher DOCN ratios in the leachate within the gap, compared to the nongap area (Table 3). The water samples collected from beneath and around the fallen logs showed DON values similar to those found in the gap area while the DOC values were much higher (Table 4). The PRS™‐probes did not give any conclusive information with respect to inorganic N (Table 5).

**Table 2:** Soil properties surrounding each lysimeter site divided according to nongap area and gap area

	*N*	Tot‐C (g/100 g)		Tot‐N (g/100 g)		NO_3_–N (μg/kg)		NH_4_–N (mg/kg)		CN ratio	
		Mean	*SD*	Mean	*SD*	Mean	*SD*	Mean	*SD*	Mean	*SD*
Nongap	8	49.54	2.03	1.73	0.27	180.9	63.56	5.32	4.40	29	5
Gap	8	50.96	1.13	1.86	0.26	215.1	47.23	5.55	3.04	28	4

	*N*	pH		Ca (g/kg)		Mg (g/kg)		K (g/kg)	
		Mean	*SD*	Mean	*SD*	Mean	*SD*	Mean	*SD*
Nongap	8	3.95	0.29	0.35^a^	0.21	0.17	0.09	0.51	0.13
Gap	8	3.76	0.12	0.66	0.30	0.26	0.07	0.47	0.15

a Significant differences (α 0.05).

**Table 3:** Comparisons between soil water extracted from nongap area and gap area, average of seven sampling occasions distributed throughout the growing season

	*N*	DOC (mg/L)		DON (mg/L)		NO_3_–N (mg/L)		NH_4_–N (mg/L)		DOCN ratio	
		Mean	*SD*	Mean	*SD*	Mean	*SD*	Mean	*SD*	Mean	*SD*
Nongap	8	42.13	31.75	0.56	0.23	0.012	0.005	0.056	0.062	60^a^	10
Gap	8	48.57	16.62	0.69	0.22	0.012	0.003	0.149	0.220	73	13

	*N*	pH		Ca (mg/L)		Mg (mg/L)		K (mg/L)	
		Mean	*SD*	Mean	*SD*	Mean	*SD*	Mean	*SD*
Nongap	8	4.19	0.28	0.21	0.19	0.11	0.08	0.34	0.24
Gap	8	4.03	0.27	0.69	0.60	0.22	0.19	0.43	0.58

a After mean values indicate significant differences (α 0.05).

**Table 4:** Properties of soil water samples extracted by macro–rhizon–lysimeters from the upper 10 cm of the soil in different distances from two decomposing stems in the gap area, at six different occasions during the growing season

Distance from stem	*N*	DOC (mg/L)		DON (mg/L)		NO_3_–N (mg/L)		NH_4_–N (mg/L)		DOCN ratio	
		Mean	*SD*	Mean	*SD*	Mean	*SD*	Mean	*SD*	Mean	*SD*
Directly under	2	92.6	61.4	0.67		0.01	0.00	0.10	0.08	75	
10 cm to each side	4	48.7	16.8	0.64	0.16	0.01	0.00	0.06	0.01	22	77
90 cm to each side	4	53.5	22.8	0.63	0.21	0.01	0.00	0.05	0.03	70	12

Distance from stem	*N*	pH		Ca (mg/L)		Mg (mg/L)		K (mg/L)	
		Mean	*SD*	Mean	*SD*	Mean	*SD*	Mean	*SD*
Directly under	2	3.99		0.75		0.26		0.14	
10 cm to each side	4	4.12	0.12	0.64	0.14	0.21	0.02	0.32	0.10
90 cm to each side	4	4.07	0.22	0.40	0.10	0.19	0.05	0.50	0.49

**Table 5:** Comparisons between PRS‐probes in nongap area and gap area, PRS(tm)‐probe supply rate given as μg/10 cm^2^ over 110 days

Forest	*N*	μg/10 cm^2^ over 110 days									
		NO_3_–N		NH_4_–N		Ca		Mg		K	
		Mean	*SD*	Mean	*SD*	Mean	*SD*	Mean	*SD*	Mean	*SD*
Nongap	8	6.8	2.7	9.1	2.3	349.7^a^	70.7	183.5^a^	60.1	692.5a	264.2
Gap	14	10.6	7.7	8.6	2.7	770.3	330.4	267.1	94.1	124.2b	90.5

a After mean values indicate significant differences (α 0.05).

The soil samples show that there was significantly higher Ca concentration in the gap area; also the Mg concentration was higher though not significant (Table 2). The soil water samples also show higher, not significant, Ca and Mg concentrations in the gap (Table 3). For the PRS™‐probes, both the Ca and Mg supply rates were significantly higher in the gap (Table 5), while K supply rates were significantly higher in the nongap area. No differences in K concentrations were obvious in the soil or soil water samples. The concentrations from soil water extracted from directly under the gap‐maker trunks showed high values of Ca and Mg and low values of K (Table 4).

## 4 DISCUSSION

### 4.1 Stand and gap dynamics

The long period of stable aboveground biomass accumulation measured in the six permanent plots in the small old‐growth stand at Karlshaugen indicates a steady‐state biomass and an age‐related decline in aboveground biomass. This indication of steady state might be of limited value because of the small number of grid plots and the small size of the stand in conjunction with no data collected from the grid plots in 1955. However, the more substantive data on the population stability of the complete stand inventory of diameter frequency distributions from 1930, 1955, 1978, and 1997 support the hypothesis of a steady state from 1955. The estimation of biomass and the analysis diameter frequency distribution seem to contradict one another for the period of close to steady state. While the population stability analysis proposes a steady state from 1955 to 1997, the biomass consideration proposes a steady state from 1930 to 1978 and until gap formation. This contradiction is due to different scales in question, but also the difference of biomass consideration versus stability consideration. The advantage of the grid plots is that we know exactly the amount of ingrowth, mortality, and survivor trees for the investigation period, but for a small sample size and area. Moreover, the combination of the data from grid plot measurements and the complete diameter analysis suggest a steady‐state phase prior to the gap formation. This work demonstrates the importance of the spatial scale, on the one hand, the gap formation did not change the complete diameter distribution for the stand, but on the other hand, a decreased biomass was observed on the permanent plot scale. Patchiness in gap formation and the small number of gap‐maker trees compared to the total number of trees in the stand is probably the main reason for this. The idea of the steady state is still controversial but useful for describing a stand undergoing a slow long‐term change (Borman & Likens, 1979). Age‐related decline in biomass production is regarded as fundamental for understanding forest growth and for global carbon budgets (Binkley et al., 2002; Stape, Ryan, Barnard, & Fownes, 2002; Gower, McMurtrie, & Murty, 1996; Ryan, Binkley, & Fownes, 1997). The first decrease in DBH of living trees within the grid plots, due to the death of larger trees, was observed in 1997 and continued to 2013 (Figure 4). The relatively long period of 50 years between the first two measurements made the recording of dead trees in 1978 uncertain. Many dead trees were snapped, and the stems were given the same DBH values for 1978 as in 1930. DBH of standing dead trees measured in 1978 also showed lower values compared to 1930 because of shrinking. This explains the relatively small DBH for standing dead trees in 1978. The median DBH of dead trees in 1997 equaled the median DBH of living trees, while in 2013, the median DBH of dead trees exceeded the median DBH of living trees (Figure 4).

The 1997 revision of the grid plots shows a decline in volume and AGB of living trees of some 30% compared to 1930 (Table 1). This decline demonstrates small‐scale synchronous mortality after a long period of steady state in the late‐successional stage. The decline in living biomass also resulted in an increase in woody detritus. According to Wirth and Lichstein (2009), decline in AGB in old‐growth forest is rare and they explain this rarity by the “shifting trait hypothesis” (Shugart & West, 1981) and buffering of regeneration. However, old‐growth forests stand where Norway spruce established after severe disturbances tend to be single cohort and single‐species stands (Sirén, 1955). This probably makes them more disposed to age‐related biomass decline than uneven‐aged mixed old‐growth forests.

Gap size is an important characteristic, but the upper size limit of what constitutes a gap is still debated (Hytteborn & Verwijst, 2014; Schliemann & Bockheim, 2011). A size distinction between gap and patch for boreal forest of lesser or greater than 200 m^2^ has been proposed, but irregular geometry and open spaced mountainous forest make precise definitions difficult. The studied gap size of 1,161 m^2^ is within the range (10–5,000 m^2^) of what is reported by others (Schliemann & Bockheim, 2011). Actually, the size is close to the median patch size of 1,200 m^2^ observed after impact of drought and bark beetle (*Ips typographus*) in the Arkhangelsk region in Russia (Kuuluvainen et al., 2014). The observed gap is an irregular “Donald” shaped polygon. Irregular‐shaped gap‐ or patch‐polygons are common (Schliemann & Bockheim, 2011), but also a challenge for evaluating the ecological impacts of gap size. Gap size often increases with time because of wind and gap expansion has been shown to be more frequent than gap initiation (Worrall et al., 2005). The ten dead spruce trees outside the gap are the outer trees killed by bark beetles. Bark beetles are shown to have short dispersal distances (Wermelinger, 2004).

Gap initiation caused by bark beetles is difficult to predict because it is a function of both stand conditions and beetle behavior (Okland, Nikolov, Krokene, & Vakula, 2016; Stadelmann, Bugmann, Wermelinger, & Bigler, 2014). However, volume and age of host trees have been shown to be important determinants. Karvemo, Van Boeckel, Gilbert, Gregoire, and Schroeder (2014) found increasing risk for attack correlated well with increasing standing volume up to 200 m^3^/ha. At such high standing volumes, the probability for presence of susceptible trees increases. Susceptible trees in our study would be trees infested by brown rot or incur abiotic stress like repeated snow break. The role of fungi‐driven gap initiation has been emphasized (Lewis & Lindgren, 2000). Trees killed by bark beetles were larger trees. Gap‐makers were characterized by DBH > 30 cm and heights >16 m (Figures 6 and 7). In this field study, we document the process of gap initiation and gap formation, but with lack of replications. True replication under natural conditions is complicated (Ryan, 2011). Within the small intact area, we have so far just observed one gap formation during the last 87 years. More gaps would have allowed replication, but heterogeneity in the forest ecosystem would restrict true replication. We still think that our observations are important for understanding the role of gap formation for the site condition and disturbance regime in question.

Downy birch, rowan, salix spp., aspen, Scots pine, and Norway spruce probably dominated the early succession stages of the stand. During a directional change, broadleaves and Scots pine have been excluded from the stand by shading of Norway spruce, which is common in Fennoscandia (Engelmark, 1987; Sirén, 1955). The mean age of the spruce stand was 175 years at breast height (Figure 8) while the fire took place 255 years ago, which might indicate 80 years for reaching breast height. Suspended regeneration is well documented from boreal old‐growth forests (Haagvar & Tveite, 2011; Kuuluvainen, Maki, Karjalainen, & Lehtonen, 2002; Shorohova, Fedorchuk, Kuznetsova, & Shvedova, 2008). The stand probably established after a few good seed years, which can be infrequent for Norway spruce. Suspended regeneration from the fire polygon (Figure 1) also indicated that reaching breast height (1.3 m) may take 80 years (Figure 10). However, as the stand was established after fire, the period from seed to breast height was probably shorter, but 40–60 years seems reasonable. The national forest inventory of Norway (NFI) estimated time to breast height for spruce to 31 years on middle site index for the same county in 1939 (NFI, 1941). Two hundred years old Norway spruce trees close to the gap edge showed increased radial increments after gap formation demonstrating that old trees still have capacity for increased carbon sequestration (Figure 9).

The amount of downed coarse woody debris (CWD) based on the grid plots reached 106 m^3^/ha (Table 1) which is six times larger as compared to mature managed spruce dominated forests in Norway (Storaunet & Rolstad, 2015). The CWD of 240 m^3^/ha in the gap area demonstrates the importance of gap formation for spatial variability of CWD at the stand level. The amount of CWD in our study corresponded very well with volumes of CWD from 100 to 150 m^3^/ha as reported from mesic old‐growth forest from hemi boreal and southern boreal zones (Siitonen, Martikainen, Punttila, & Rauh, 2000). The bilberry dominance in the ground vegetation in the gap area has been remarkably stable since 1930 (Figure 2) (Nygaard & Odegaard, 1999) reflecting that the light conditions in the gap area have not led to major changes in ground vegetation.

### 4.2 Belowground disturbance

The impacts of gap formation on soil nutrients in the forest floor 20 years after gap initiation were most pronounced for calcium. Biogeochemical cycling of Ca probably exerts more control on both the structure and the function of forest ecosystems in particular for later successional stages than earlier recognized (McLaughlin & Wimmer, 1999). Ca conservation will be more important in late‐successional stages as availability becomes more limited. Exchangeable Ca in the O horizon and plant–root simulator (PRS) supply rates of Ca were both significantly higher within the gap compared to the nongap area (Tables 2, 3, 4). Calcium has been shown to accumulate in foliage up to time of senescence and constitute a major component of permanent plant tissue, and litter fall play an important role in Ca cycling (Johnson, 1992). The increase in CWD as a Ca pool together with a decreased uptake after gap formation has increased exchangeable Ca in the gap area compared to the nongap area. Ca release from decomposing CWD can explain this long‐lasting stabilizing effect. Johnson, Todd, Trettin, and Mulholland (2008) noted increased exchangeable Ca as an exception after tree mortality in their study of decadal change in Calcium from Walker Branch Watershed. The increase in exchangeable Ca is also supported by our fine‐scale study of leachate under decaying fallen trees.

This is consistent with other studies of soil leachate under CWD (Bantle et al., 2014; Hafner, Groffman, & Mitchell, 2005; Lajtha et al., 2005; Spears & Lajtha, 2004). Several studies also show increasing nutrient release under well decomposed CWD than under fresh CWD (Brais & Drouin, 2012; Laiho & Prescott, 2004; Morris, Wiebe, Luckai, & Reid, 2015). Palviainen et al. (2010) studied decomposing stumps of pine, spruce, and birch and found that loss of Ca and Mg increased gradually and was highest after 20–40 years which fits well with the results in our study. The supply rate of both Ca and Mg to the PRS™‐probe was significantly higher in the gap area compared to the nongap area. Additionally, dominance by the clonal bilberry may contribute to Ca conservation at ecosystem level because of the strong tendency for Ca accumulation (Ingestad, 1973; Nygaard & Abrahamsen, 1991). A different pattern was seen in K where a larger supply rate was found in the PRS™‐probes in the nongap area as compared to the gap area. Palviainen et al. (2004) studied nutrient release from logging residue of Norway spruce, Scots pine, and silver birch and found that the K release and leaching follow the decomposition of needles and bark and is rapidly lost. In their study, almost 90% of the initial amount of K, but only 8% of the initial Ca, was leached within 3 years. Twenty years after gap formation, we did not find any difference in K leached in the soil water between the gap and nongap area. Neither did the fine‐scale investigation of the leachate under the decaying tree logs show any elevated K concentration, suggesting that most of the mobile and easily released K already was leached. The higher K supply rates in the nongap area are also influenced by throughfall. Long‐term monitoring near Karlshaugen showed low concentrations of base cations in rainfall, but elevated concentrations of K in the throughfall in spruce forest (Moffat, Kvaalen, Solberg, & Clarke, 2002).

Soil carbon and nitrogen did not show any differences between the gap and nongap areas. This could partly be due to sampling procedure as CWD was excluded from the soil samples. The dried samples were sieved, and only the fraction <2 mm was analyzed. Any coarse woody fragments with high C and low N content would therefore have been removed before analysis. The fine earth fraction would consist of more decomposed organic material with lower C content and therefore show little difference between the gap and the nongap area with regard to C and N concentration. Soil water from the gap showed a significantly higher DOCN ratio compared to the nongap area, possibly reflecting the influence of the CWD. The fine‐scale analysis of leachate from under decomposing gap‐maker logs showed twice as much DOC under the logs compared to leachate 10 cm and 90 cm from the log (Table 5). This high DOC was not followed by equally elevated DON concentrations explaining the high DOCN ratios found under the decaying log and in the gap area in general. This was consistent with other studies of leachate from decaying CWD. (e.g., Lajtha et al., 2005; Morris et al., 2015).

We found no significant differences between the gap and nongap area with respect to inorganic nitrogen; neither in soil, soil water, or the PRS™‐probes. Nitrogen dynamics in gap areas are complex, and studies have shown diverging results. Prescott (2002) suggested that canopy gap formation would increase soil N availability due to elevated N mineralization rates. Bade, Jacob, Jungkunst, Leuschner, and Hauck (2015) came to a different conclusion in their study of N mineralization in an old‐growth spruce forest. They found that the N mineralization was lower in the more open patches than in the closed forest. They explained this as a result of reduced litter supply, lower canopy N interception, and less drained soils due to reduced canopy respiration. Palviainen, Laiho, Makinen, and Finer (2008) found that decomposing CWD retained N rather effectively in boreal forests. Within their study, 40% of initial N was released from pine and spruce within 30 years. However, gap edge trees showed increased growth and uptake after gap formation in our study (Figure 9). This increased uptake, combined with an irregular gap shape, has probably reduced differences in limiting nutrients like N between the gap and nongap areas. This may indicate that an aboveground gap does not necessarily create a belowground gap which has been shown for experimental gaps (Cambell, Fineer, & Messier, 1998) or that the belowground gap closes faster.

## 5 CONCLUSION

Our long‐term study from Karlshaugen demonstrates gap dynamics after more than 50 years of steady state and a multiscale disturbance regime in an old‐growth forest. The observed disturbance dynamic on different scales in time and space produced higher aboveground and belowground heterogeneity in habitats and coarse woody debris, but also spatial and temporal gradients in Ca supply. Our study of the nutrient levels of the forest floor suggests that natural gaps of old‐growth forest provide a long‐lasting biogeochemical feedback system for nutrient cycling particularly with respect to Ca and probably also N providing conservation of nutrient supply. The old‐growth stand shows longevity of natural Norway spruce forest exceeding the rotation period practiced by forestry by about 100 years before gap formation. Two‐hundred‐year‐old Norway spruce trees close to the gap edge respond with high plasticity to changed growth conditions showing the importance of the edge zone as hot spots for establishing heterogeneity, but also a potential for carbon sequestration in old‐growth forest. In order to mimic a long‐term natural disturbance regime, repeated clear‐cutting on the same area should be avoided and replaced by a more diversified management strategy.

## CONFLICT OF INTEREST

None declared.

## AUTHOR CONTRIBUTION

P.H. Nygaard, L.T. Strand, and A. O.Stuanes performed fieldwork. P. H. Nygaard and L.T. Strand processed and analyzed vegetation and soil data and wrote the manuscript.
